# Grouse Disease

**Published:** 1904-08-20

**Authors:** 


### The Hospital.

A Journal of

The Medical Sciences and Hospital Administration.

Vol. XXXVI.—No. 934. AUGUST 20, 1904.

### GROUSE DISEASE.

The account recently given by "a correspondent" in the *Times* paper of the doings of Lord Onslow's Commission or Committee for the investigation of Grouse Disease furnishes a curious example of the way in which questions of pure science are dealt with in this country. The economic importance of the bird, and the fact that for nearly a century past its numbers have been periodically diminished by an epidemic peculiar to its species, furnish excellent reasons for undertaking the inquiry, and reasons still more weighty for committing it to persons who might be capable of carrying it to some trustworthy conclusion. We are, nevertheless, informed, with charming *naïveté*, that Lord Onslow has actually been taxed with neglecting the scientific side of the question by composing his Committee of "sportsmen rather than of pathologists," a composition which seems to us fairly comparable to the appointment of a committee from the nursery to decide upon the correct interpretation of a Hittite inscription. We are further told that Dr. Klein, in 1887, went to Scotland in order to investigate the cause of the disease, and that he discovered a bacterium to which he believed that it might be attributed. Moreover, by making cultures from the blood of grouse dead from the disease he succeeded in communicating a similar malady to other creatures, but not to healthy grouse, and he thus just failed to prove that he had really discovered what he sought. But "the trend of science" points to the probability that he was right, and that, if he was not successful in finding the specific microbe required, nevertheless bacteria "of some kind" are the primary cause of the disease. If this be so, a great number of "old theories" (presumably sportsman's English for "guesses") "will have to be abandoned"; and it is comforting to hear that "no doubt the Committee will call for the evidence of pathologists if it is required." We are assured, however, that "any knowledge based upon the discovery of a specific microbe in the blood or organs of diseased grouse could only be useful under very circumscribed possibilities. We could not, for instance, administer hypodermic injections to the wild grouse, even if bacteria were proved to be the first cause, and if cultures from them could be rendered either remedial or preventive." Pathologists, we feel assured, will prostrate themselves before the sagacity which has made such a discovery as this, and which has evidently pictured to itself processions of men of science strolling over the moors with "cultures" and injection syringes in their pockets, and vainly inviting the grouse to submit themselves to the operation by which they would be protected against a contagion so fatal to their kind. We see no inherent impossibility in the suggestion that grouse may be more rational than anti-vaccinationists; but we fear that some generations of education would be required before they would come for injection in sufficient numbers to afford them any large measure of safety.

It is painful to read, in a further paragraph of the communication to which we refer, that when the "late Dr. Pasteur" had consented to examine into the cause of the grouse disease, not a single bird was sent to him. We may recall that this same "late Dr. Pasteur," whose discoveries in the direction of curing or preventing silk-worm disease, vine-disease, anthrax, fowl cholera, and the causes of imperfect fermentation, were of sufficient pecuniary value to his country to provide the means of paying the German war indemnity, published at Lyons, while the war was in progress, a pamphlet with the title "Pourquoi la France n'a pas trouvé d'hommes supérieurs au moment du péril," and that the answer which he gave to the inquiry was based upon "l'oubli, le dédain que la France avait eu pour les grands travaux de la pensée, particulièrement dans les sciences exactes." Can we not conceive the scorn which he would have poured upon Lord Onslow's "sportsmen" gravely speculating concerning problems the very first conditions of which would be utterly unknown to them, and inviting the co-operation of "practical men" not one of whom would ever have seen a bacterium in his life, or would be able to recognise it if by any chance it came under his view? It is abundantly obvious to all who have any knowledge of kindred questions, or of the way in which complete or approximate solutions of them have been obtained, that Lord Onslow's Committee of Sportsmen will accomplish nothing beyond a waste of time and money, and that, even with the full intention of calling for the evidence of pathologists if it is required, they are practically certain to fail in recognising the occurrence of an opportunity leading to the requirement. "Who shall say," asked Faraday, speaking of philosophers, "that new knowledge is not under our eyes every day, and yet suffered to pass away unrecognised?" And nothing can be more certain than that the "practical men" (those wildest of all speculators, because their speculations are not under the restraint of knowledge) will miss the very chances of discovery which may be op·n to them. In relation to the sources or the propagation of disease they would be illuminated only by the light of nature, and their ultimate conclusions wou'd be as useful as those of Mrs. Gamp about puerperal fever, or of old women about rheumatism. Solomon has told us the character of those who are wiser in their own conceit than seven men who can render a reason, and the description seems not inapplicable to amateurs who set themselves to form conjectures about methods of arresting the spread of an infectious malady. We can only paraphrase the speech of Dutch William when he touched a man for the "King's evil," and wished him better health and more sense. May the former be the good fortune of the grouse, and may the latter come in time to the rescue of the Committee!

